# Correction: Lloret et al. A Data-Driven Framework for Digital Transformation in Smart Cities: Integrating AI, Dashboards, and IoT Readiness. *Sensors* 2025, *25*, 5179

**DOI:** 10.3390/s25195938

**Published:** 2025-09-23

**Authors:** Ángel Lloret, Jesús Peral, Antonio Ferrández, María Auladell, Rafael Muñoz

**Affiliations:** 1 Language and Information Systems Group, Department of Software and Computing Systems, University of Alicante, 03690 Alicante, Spain; lloret@ua.es (Á.L.); antonio@dlsi.ua.es (A.F.); maa104@gcloud.ua.es (M.A.); rafael@dlsi.ua.es (R.M.); 2 Lucentia Research Group, Department of Software and Computing Systems, University of Alicante, 03690 Alicante, Spain

There was an error in the original publication [1]. Reference [72] was incorrect. The original Ref. [72] has been replaced by a new Ref. [72] (Nižetić et al., 2020). The authors state that the scientific conclusions are unaffected. This correction was approved by the Academic Editor. The original publication has also been updated.

72. Nižetić, S.; Šolić, P.; López-de-Ipiña, D.; Patrono, L. Internet of Things (IoT): Opportunities, issues and challenges towards a smart and sustainable future. *J. Clean. Prod.* **2020**, *274*, 122877. https://doi.org/10.1016/j.jclepro.2020.122877.
